# Iranian women’s experiences of infertility: A qualitative study

**DOI:** 10.18502/ijrm.v18i1.6203

**Published:** 2020-01-27

**Authors:** Nader Aghakhani, Béatrice Marianne Ewalds-Kvist, Fatemeh Sheikhan, Effat Merghati Khoei

**Affiliations:** ^1^Inpatient Safety Research Center, Urmia University of Medical Sciences, Urmia, Iran.; ^2^Department of Psychology, Stockholm University, Sweden.; ^3^Department of Psychology, University of Turku, Finland.; ^4^Department of Midwifery, Khalkhal Branch, Islamic Azad University, Khalkhal, Iran.; ^5^Brian and Spinal Cord Injury Research Center (BASIR), Neuroscience Institution, Tehran University of Medical Sciences, Tehran, Iran.; ^6^Community Based Participatory Research Center, Iranian Institute for Reduction of High-Risk Behaviors, Tehran University of Medical Sciences, Tehran, Iran.

**Keywords:** Iran, Women, Infertility, Qualitative.

## Abstract

**Background:**

There are concerns and diverse experiences related to infertility and childlessness. The lived experience of infertile people from various cultures needs to be explored.

**Objective:**

The aim of this qualitative study was to explore Iranian women experiences of their infertility.

**Materials and Methods:**

The data comprised interviews about fertility issues in the Persian language with eighteen women, aged 17-45 yr old, who agreed to be interviewed at the Mottahari Infertility Treatment Clinic, affiliated to the Urmia University of Medical Sciences about their fertility problems. They were approached by the researchers at the time of their first visit. The verbatim transcribed interviews were analyzed using deductive conventional content analysis.

**Results:**

The experiences of the informants were conceptualized into four major themes: 1) Shock (subthemes: Disbelief and Denial); 2) Reaction (subthemes: Distress, Guilt, Loss of self-esteem and Sexual reluctance); 3) Processing (subthemes: Internal processing, Avoidance, Marriage at risk, External processing, Stigma caused by the family and Stigma caused by the community) and 4) Reorientation (subthemes: Forgetting, Marriage to saving marriage and Sexual consent).

**Conclusion:**

Infertility can be a challenging condition. Considering that infertility-related issues affect Iranian women more contextual factors is necessary. So, culturally sensitive and gender specific protocols are suggested to provide suitable and about culturally sensitive and gender-specific protocols is a necessity in order to provide suitable care to infertile women.

## 1. Introduction

It is estimated that 72.4 million women currently experience infertility; of these, 40.5 million are seeking infertility treatment. Yet prevalence estimates of lifetime infertility fluctuate broadly because there is no reliable definition of infertility and samples are of different ages and measured using varied methods (1), concurrent with several medical, psychological and social problems including frustration, depression, anxiety, hopelessness, guilt, and feelings of worthlessness in life (2, 3).

The inability to have a child is frequently regarded as a personal tragedy, having negative impacts on the entire family as well as the wider local community (3). The evidence demonstrates that childbearing ability is often the only way for women to enhance their status in the community in developing countries in particular, where the nature of social interactions with regard to fertility is highly gendered. In other words, pregnancy and childbirth both are perceived as women' feminine potentials (4, 5).

In this qualitative inquiry, we aimed to understand Iranian women's experiences of infertility. We have explored their perceptions of childlessness by means of deductive conventional content analysis. As the unit of analysis, we employed the trauma-progression scheme outlined by Akizuki and Kai (6). The scheme integrates the four stages of a trauma experience: shock, reaction, processing, and reorientation.

## 2. Materials and Methods 

In this qualitative study, we obtained informed consent from the informants, who entered the study voluntarily. Confidentiality principles were explained and validated.

We approached the informants if they are diagnosed infertile by a professional team in the selected infertility treatment centre, in Urmia. These participants had history of primary or secondary infertility as well as those who were candidates for egg donation. Each interview began with a warm-up question followed by open-ended questions that assessed of their experience of childlessness. The questions were as follows: “Why do you want to treat your fertility? How does being infertile feel? The interview continued with probing questions. The interviews were 45-120 minute long and were recorded. The data comprised 18 interviews in the Persian language. Semi-structured face-to-face personal interviews were used for data collection.

**Figure 1:**
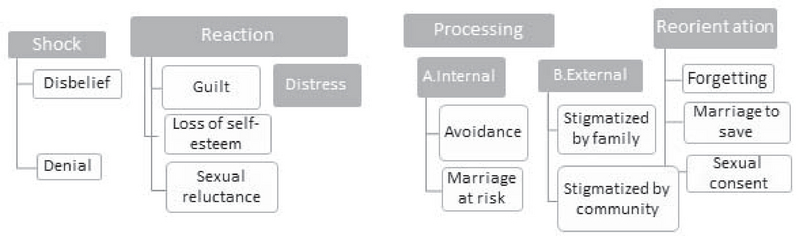
Main Themes and Subthemes about the Participants' experiences with infertility.

An experienced nurse was considered a proper and valid instrument to conduct interviews. She was able establish rapport with couples and women in particular. All interviews were transcribed verbatim in the Persian language. Each interview was analyzed by means of deductive conventional content analysis. A total of 648 initial codes were listed; they were clustered into four trauma processing themes, which were further divided into 13 sub-themes, as shown in Figure 1. To determine the data trustworthiness, Lincoln and Guba's criteria of credibility, transformability and confirmability for maintaining the trustworthiness of the study findings were used (7).

### Ethical consideration

The research was conducted according to the Declaration of Helsinki and was approved by the Ethics Committee of Urmia University of Medical Science (code: IR.umsu.rec.1396.320). The participants learned about the objective and were asked to sign an informed consent.

### Statistical analysis

Data collection and data analysis were performed concurrently. The data were analyzed using Graneheim and Lundman's content analysis approach (8). First, interviews were transcribed verbatim. Transcripts were read three times to times to gain a complete understanding of their content. Codes were compared and sorted into new categories according to similar characteristics. Finally, categories were compared with each other and changed into higher-level main categories. MAXQDA 10 software was used for managing the coding and categorizing and for linking the research interviews to the codes.

## 3. Results

Participants' characteristics and infertility-related issues are shown in Table I. Four major themes emerged: shock, reaction, processing, and reorientation. Women's processing phase relative to learning about their infertility is described by two distinct paths: internal and external processing; the families of the women and their husbands were the seminal elements that shaped external processing (Figure 1).

### Shock

The majority of women experienced `shock' after learning about infertility. This experience was described in two ways: disbelief and denial.

### Disbelief

The majority of women questioned their identity as a feminine individual. From their viewpoint, reproduction was an important factor in believing in themselves. One of the participants expressed her confusion through this statement:

“After marriage, every woman thinks that she should be a mother as soon as possible, but I could not have a baby. I thought that I was an atypical woman, but I could not believe that `I was a woman”.

### Denial

`I told myself that it cannot be true; I have to have a child anyhow as soon as possible'

`When the doctor told me that I was infertile and could not have a child, I said how dare you say that I cannot have children? You are a liar.'

### Reaction 

Participants reported that childlessness is a curse. Any marriage without a child is fertile ground for separation.

### Distress

`Why should I put up with my husband's family's blame? I cannot even do anything. This issue always worries me. O, my God! I cannot imagine what has befallen me!'

### Guilt

`I had sex with my husband. He told me that we should not have a child early in our life. Unfortunately, I got pregnant, and he forced me to have an abortion. I became infertile after that induced abortion.'

### Loss of self-esteem

`When a woman understands that she is fruitless, she thinks of herself as inadequate and loses her self-esteem'.

### Sexual reluctance

The ultimate marital dream of the majority of the participants is to have children; women in this study reported unhappiness in their life and several of them reported reduced sexual activity with their husbands. Consequently, with the recurring failure in their attempts to bear children, their desire to have intercourse had altered:

`My husband has not had sex with me for more than two months because he thinks it is a waste of time. This frightens me so much. I do not want to have sex with him either. He does not love me and has sex with me like an obligation without any love. My married life is at risk because this situation will culminate in divorce.'

## 4. Processing

### Internal processing

The reality sinks in; denial no longer functions as a defense against their reality:

`I desired to have children very much and was really happy after finding out that I was pregnant. But I had to abort my child when fetal abnormalities were diagnosed at the gestational age of 5 months; I will be sad for the rest of my life. O, my God! why me?'

### Avoidance

The condition of infertility awakens many difficult feelings, such as anger. Managing the avoidance of difficult feelings serves as a defense as well as coping mechanism at the same time:

`I do not like to go anywhere and like to be alone at home. A woman who does not have a child must die!'

### Marriage at risk

A woman who is not able to give her husband a child faces the danger of being divorced. This fear is real while she is subjected to psychological abuse; her husband threatens and blames her.

`When we knew about my infertility after our marriage, we thought of divorce. I will not enjoy living this kind of useless life.'

### External processing

Any of the participants were excluded from their roles in the family and the wider community*. *They felt devalued by their husbands, his family members, and the community. They reported instances of humiliation and blame from their relatives. Women with a lower educational and socioeconomic level were at a higher risk of being subject to psychological abuse; not being able to bear a child, preferably a son, is considered a horrified problem because it is perceived as causing the extinction of the family lineage.

### Stigma caused by family

Several participants reported that they are blamed for being infertile; the women have been subjected to negative experiences, giving rise to the feeling that they will feel secure only by bearing a child. They have been subjected to psychological abuse in the following forms: `My husband always humiliates me and my family for my infertility. He even told me that one day he will divorce me'.

### Stigma caused by the community 

For most participants, infertility has resulted in negative consequences in their social context. They preferred not to attend social events as a strategy to cope with the stigma of infertility and to avoid awkward questions from the community.

'I do not like to attend my husband's family events. If I have to be there, I do try to stay in the background”.

### Reorientation

The participants struggle to look to the future and to regain confidence in their lives.

`I think women need to do something with their nurturing spirit. Because I don't have children, I have had more time to take care of other people. Why should we be concerned with what our family tells us?'

### Forgetting

Some of the participants try to move past their fertility problems by finding a new hobby or job.

`My husband and I try to work hard and forget our problem. We do not let other people judge our marriage or let them tell us what we should do.'

### Saving the marriage 

Some couples love each other and accept the reality, even tragic phenomenon like childlessness. They do happily stay together.

`When we know about our fertility issue, it makes us nervous, and we get disappointed. We have to choose between getting divorced and having a good life, and we have chosen the latter alternative. Why not?

### Sexual Submission

Some of the participants tried to find a solution for the problem employing sexual submission.

“I enjoy being a good wife in the bed. I mean that I want to have a good sexual relationship with him and be a good housekeeper in his home”.

**Table 1:** Participants' characteristics and infertility-related issues


**Characteristics**	**n (%)**	**Mean ± SD**
Age (17-45 yrs)	18 (100)	28.11 ± 9.2
Education		
College student or above	2 (11)	
High school or diploma	5 (28)	
Secondary school	3 (17)	
Primary school	8 (45)	
Infertility		
Primary	11 (61)	
Secondary	7 (39)	
Duration (1-25 yrs)	18 (100)	7.5 ± 6.5
Treatment (1-22 yrs)	18 (100)	6.9 ± 5.67
Causes of infertility		
Endometriosis	8 (44)	
Uterus dysfunction	5 (28)	
Habitual abortion	4 (22)	
Unexplained	1 (6)	

## 5. Discussion

The present study aimed to explore the experiences of infertile Iranian women. We recognized four stages corresponding to experiences of shock, reactions, processing, and reorientation as outlined by Akizuki (6). Supporting our findings, it is has been reported that childless women are at a greater risk of emotional disturbance, lower quality of life, marital discord, depression, anxiety, and posttraumatic stress disorder (9). In reacting to their infertility, women's narratives revealed fear and anxiety and self-accusation as well as sexual avoidance similar to others (10). However, another study has found that infertile women showed higher rates of alexithymia compared to fertile women. They have difficulty talking about their predicament if the other part is unwilling to listen or if even the people closest to them are judgmental and rejecting (11). In terms of the processing phase, our participants used avoidance both as a defense and as a coping mechanism for difficult feelings. Akizuki and colleagues have highlighted that women undergoing fertility treatment socialize themselves in accord to their peers during their treatment at the hospital. One will be dramatically disappointed at the time of another woman's success and would feel as a `loser' (6). Based on our findings, we argue that Iranian women are subjected to humiliation and isolation by both family members and the community and have to cope with different forms of embarrassment. In other side of the coin, infertile women are supported emotionally by members of the community; however the participants' narratives highlighted women's negative perception of other's empathies.

##  Limitations

One of the limitations of the present study is that we have used a small sample size of women with fertility problems who belong to a culture in which they would be expected to give birth to a child in order to gain their place in society. The results were derived from patients' self-reporting while still on infertility treatment. Their memory can, therefore, be distorted. They were also in different stages of trauma processing, and only a few of them showed signs of having accepted their fertility problem. Altogether 39% suffered from secondary infertility, which means that they had already been through traumatic experiences; this would have certainly added to their present situation (12).

##  Conflict of Interest 

The authors declare that there is no conflict of interest.
